# Sternectomy for Treating Advanced Non-Melanoma Skin Cancer

**DOI:** 10.1155/2019/3948782

**Published:** 2019-05-30

**Authors:** Victor Emmanuel Gadelha Pinheiro, Bianca Rohsner Bezerra, Luís Arthur Brasil Gadelha Farias, Irapuan Teles de Araujo Filho, Marcio Ribeiro Studart da Fonseca

**Affiliations:** ^1^Ceará Cancer Institute, Fortaleza, Ceará, Brazil; ^2^Faculty of Medicine, Federal University of Ceará, Fortaleza, Ceará, Brazil

## Abstract

**Introduction:**

Skin cancer is a rare indication of sternectomy. Our goal is to report the clinical course of seven patients who underwent sternectomy for skin cancer.

**Methods:**

The survey data were collected from medical records of patients treated between 2008 and 2018 at Ceará Cancer Institute.

**Results:**

All patients had prolonged sunlight exposure and average disease time of two years and age of 60 years. Most patients recovered favorably after treatment with prolonged survival.

**Conclusion:**

Sternectomy remains an option with curative purposes for locally advanced skin cancer.

## 1. Introduction

The sternum is an unusual anatomical site for cancers, making sternectomy for oncologic resections rare [1]. The main indication of sternectomy is surgical treatment of infectious complications after cardiovascular and thoracic surgery, which involves a transsternal approach. Consequently, its approach, surgical goals, and reconstruction techniques are sometimes quite different from sternectomy realized in oncology. Primary or secondary neoplasms of the sternum tend to grow and infiltrate the layers of the chest wall, requiring extensive resections to achieve reliable safety margins [2].

Primary tumors of the sternum account for about 0.5% of all primary bone tumors [2]. Chondrosarcoma is the most common cause, occurring mainly in young adult men. Among secondary tumors, skin cancer is a very rare cause of sternal involvement. Utilizing the MedLine, PubMed, and Lilacs databases, with the descriptors “sternum”, “squamous cell carcinoma”, “basal cell carcinoma”, and “combination therapy”, we found no reference linking sternectomy and nonmelanoma skin cancer.

This study reported the existence of sternectomy for non-melanoma skin cancer treated at the Haroldo Juaçaba Hospital and described the indications, complications, and survival of patients who underwent this surgery.

## 2. Materials and Methods

This is a retrospective case series study, involving patients undergoing sternectomy for treatment of nonmelanoma skin cancer at the Haroldo Juaçaba Hospital, between 2008 and 2018. The ethical committee of Ceará Cancer Institute approved this study. Clinical data was acquired from patient records. Variables including age, gender, profession, sun protection measures, and period of time from beginning of symptoms to diagnosis were recorded. All patients underwent computed tomography of the neck and thorax with contrast for preoperative staging.

Extended sternectomy was defined as when there was associated contiguous organ resection, like pericardium, pleura, ribs, clavicle, and lymph nodes. The resections were described as R0 when considered complete and with satisfactory margins (larger than 2 cm from the cancer). A R1 resection was defined as the presence of marginal margins. The reconstructions were described as the use of myocutaneous flaps, polypropylene mesh for reinforcement or with primary closure of the injury.

Postoperative complications were considered as the time to extubation greater than 24 hours and the progress to respiratory failure or pneumonia postoperatively. Presence of surgical site infection, surgical wound seroma, myocutaneous flap necrosis, or deaths in the immediate postoperative period were also evaluated.

All patients underwent follow-up every three months in the first and second years, every 6 months in the third to fifth years, and annually thereafter. Patients were evaluated for tumor recurrence, defined by perceived clinical lesions confirmed by histopathology or lesions identified by radiologic studies in typical areas of metastatic progression of nonmelanoma skin cancer, such as lymph node, lung, liver, and bones.

## 3. Results and Discussion

### 3.1. Results

All patients were male. The average age at diagnosis was 60 years, with age range from 49 to 77 years. All patients were farmers without good photoprotection (no regular use of sunscreen, hats, closed long-sleeved clothes, and passing long periods of sun exposure per day). Two cases were presented as tumor recurrence. The other had, as main complaint, the presence of an ulcerated wound in the sternal region that did not heal. One patient had papillary thyroid adenocarcinoma associated and two patients were smokers with personal history smoking higher than 10 pack-years. None of them had diabetes, arterial hypertension, personal history of radiation exposure or immunodeficiency (see Table 1).

Five patients were diagnosed of skin squamous cell carcinoma (SSCC). Two patients had basal cell carcinoma (BCC). The average time for evolution of the disease was over 2 years. Only one patient reported progress in less than six months and another reported progress between 6 months and a year.

**Figure 1:**
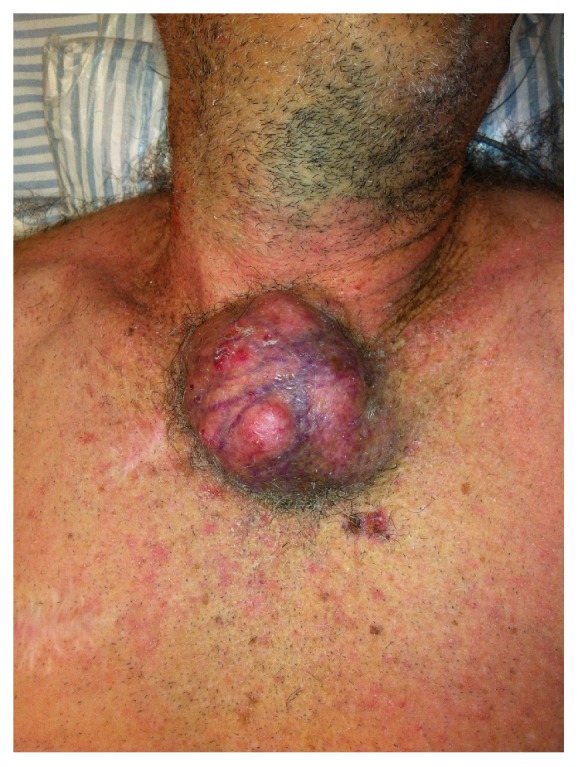
Nonmelanoma Skin cancer on sternal region, invading ribs, clavicles, and manubrium sterni.

**Figure 2:**
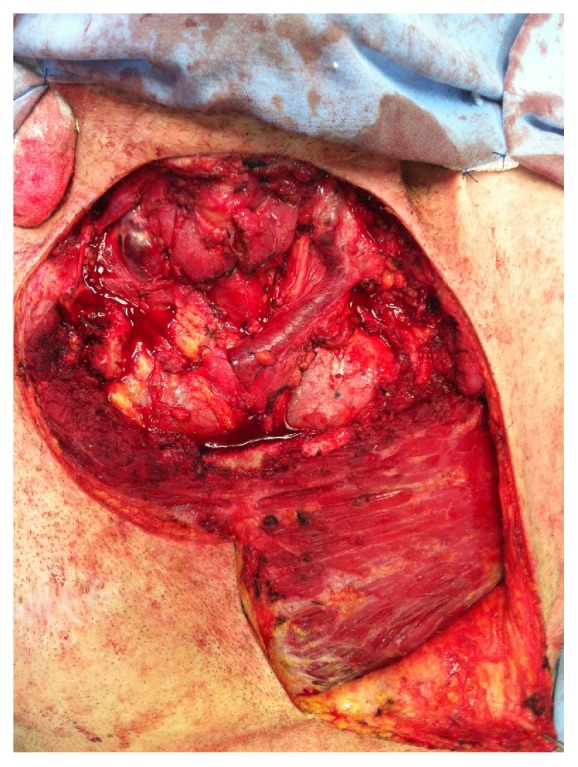
Product of extended sternectomy with resection of parts of first and second ribs, proximal area of clavicle, and manubrium sterni.

No patients underwent neoadjuvant treatment and none of them underwent total sternectomy on the same procedure. One patient had recurrence of BCC on the sternal region and underwent totalization of sternectomy four years after the first surgery. Resections had safety margins of at least 2 cm, except in one case. Six patients underwent extended sternectomy. The first three ribs were the other structures most commonly resected (see Figures 1 and 2). Combined resections of clavicles and pleura and pericardium segments were also required in two cases. R1 resection was achieved in only one patient whose histological type was BCC. The others had R0 resection. Bilateral axillary lymph node dissection, modified neck lymphadenectomy, and mediastinal lymphadenectomy were each done in one patient, respectively. Lymph node metastasis was identified in one patient with SSCC (see Table 2).

The histopathological data confirmed advanced disease, with lesions deeper than 4 cm, invasion of pectoralis muscle in all patients and sternum body in six patients. Five of them were classified as stage T4aN0M0 with the TNM staging system, one as T3N0M0, and one as T4aN1M0 (see Table 3).

All patients required reconstruction with pectoralis major muscle flap, being bilateral in one case. Polypropylene mesh helped in reconstruction in only two patients. Almost all patients were extubated in the immediate postoperative period. Only one patient had a longer intubation period (five days). The average hospital stay was 7 days, ranging between 4 and 11 days. Two cases progressed with nosocomial pneumonia. One patient developed bilateral pneumothorax, resulting from the resection of parietal pleura segments bilaterally. There was no necrosis of the myocutaneous flap. Seroma was observed in five of seven patients and was treated with needle aspiration. Adjuvant radiotherapy was performed in 4 patients due to exiguous margins; no patient received adjuvant chemotherapy.

During the outpatient follow-up, we observed a local recurrence in the chest wall in one case with SSCC one year after sternectomy. Treatment of local recurrence was carried out by surgical resection with a safety margin. One patient with BCC had recurrence on the chest wall, above the sternal body, four years later. He was treated with total sternectomy and reconstruction with myocutaneous flap. At the time this study was concluded, 6 patients were alive, and 5 were free of disease (see Table 4).

**Figure 3:**
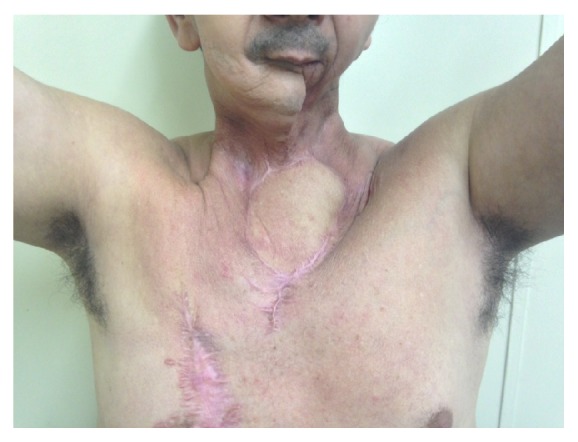
Functional preservation of the upper limbs after extended sternectomy and reconstruction with myocutaneous flap.

**Figure 4:**
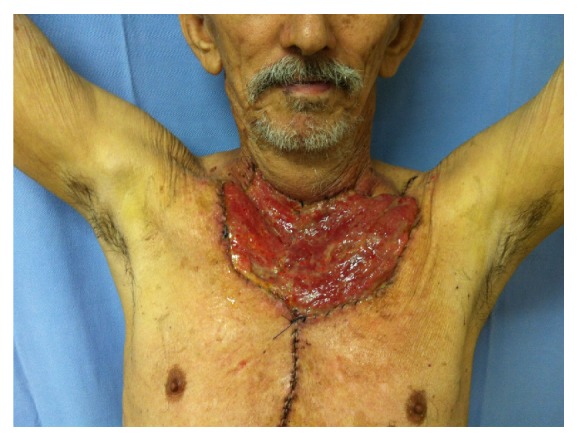
Functional preservation of the upper limbs after extended sternectomy and reconstruction with myocutaneous flap.

The average time for overall survival was 50 months. No patient had functional deficit of the movement of the upper limbs (see Figures 3 and 4).

### 3.2. Discussion

Sternectomy is a rare and complex surgery in oncology and is usually indicated for the treatment of primary sarcomas, with chondrosarcoma the most prevalent among them. Sternum resection for treatment of skin cancer is very rare. With a series of 49 cases, Butterworth et al. [1] reported that the main cause of sternectomy was involvement of sternum metastatic tumors and, secondly, sarcomas. No case with skin tumors was described among the patients, who were mostly females. Koppert et al. [2], described a further series of 68 patients, mostly female, 43 sternectomies by sarcomas, 17 by breast cancer, and 8 by sternum radionecrosis.

In Brazil, nonmelanoma skin cancer is the most prevalent tumors in both genders. It is possible there is an underreporting of this cancer, due to underdiagnosis. Consequently, estimates of incidence rates and expected numbers of new cases in relation to this cancer should be considered as minimum estimates [3, 4]. According to Brazilian National Cancer Institute, it is estimated that there will be 85.170 and 80.410 new cases of nonmelanoma skin cancer in men and women annually, respectively, by 2018 and 2019 in Brazil [3, 4]. Skin squamous cell carcinoma is the second most prevalent type, after the basal cell type. Solar radiation exposure is the main risk factor for SSCC and BCC, both more prevalent in tropical regions: 75% occur in areas around the face and neck, in fair-skinned and older people [5]. In Ceara state, in the northeast of Brazil, where solar irradiation is intense, it is not unusual for farmers to have the sternal region of their skin exposed to solar radiation for long hours, due to the cultural habit of wearing shirts, unbuttoned to the abdominal region. They live where solar radiation availability is quite elevated and ozone concentrations are naturally smaller. For these reasons, the Ultraviolet Index (UVI) observed in Brazil, as a rule, reach the highest UVI scales recommended by WHO, that is, very high (UVI between 8 and 10) or extreme (UVI higher than 11) damage to human health [6].

SSCC and BCC tend to have good prognosis when treated early. Radiotherapy could be useful for treatment of BCC or SSCC, which offers good local control, with less efficiency and more toxicity than surgery [7]. Exclusive radiotherapy is recommended when the surgery is contraindicated or as adjuvant treatment. For small and superficial lesions, topical therapies could be used, but the lesions of our patients were averagely larger than 4 cm. Under this condition, the best option for curative purposes is surgical resection.

Many factors are associated with increased risk of metastasis, such as tumors larger than 2 cm, tumors invasion beyond the dermis, anatomical location of the tumor in the lip or ear, poor differentiation, immunosuppression, perineural invasion, positive margins, local recurrence, lymphovascular invasion, and desmoplastic histological subtype [8]. Basal cell type also usually has good prognosis and chance of cure in the early stages of the disease.

With disease progression, both the BCC and SSCC can grow vertically, invading adjacent structures. In the case of presternal regions, the thinness of subcutaneous tissues facilitates sternum invasion, worsening the prognosis. However, this time for invasion is long, as seen in most of our patients, with an average disease development time of two years or more.

Resection of the tumor must be performed en bloc, with resection of skin, subcutaneous tissue, bone segments, and organs that are involved by cancer. The definition of safety margins of resection has been widely discussed. Brodland and Zitelli [9] defined safety margins for resection of SSCC as a margin of 4 mm for low-grade tumors and 6 mm for high-grade tumors. However, there is no clear definition of safety margins for bone tissues affected by this neoplasm. King et al. [10] recommend a tumor-free margin of 4 cm for highly aggressive primary tumors of the sternum and 2 cm for metastasis, or benign or low-grade malign tumors. The safety margin of 2 cm was effective in almost all patients of this study, achieving R0 resection in six of the seven cases described. Every effort must be to get three-dimensional free margins.

Complications with resections are relatively common, because of sternal anatomic relationships with important structures. Among them, the most prevalent and important is associated with the respiratory tract [11]. The structure of the musculoskeletal chest wall provides protection for the thorax and mediastinal organs and integrity for their respiratory function. Consequently, the sternum loss causes instability of the ribcage [12]. Phrenic nerve paresis or injury, resection of pleura or its opening, and rib resections worsen instability, causing atelectasis, pneumonia, and respiratory failure, which is the leading cause of death in sternectomy.

The reconstruction of the chest wall has the role to prevent or, at least, reduce the instability of the ribcage after sternal resection with or without ribs in association [13]. Muscle and myocutaneous flaps are the tissues of choice to cover the wound, reduce the risk of infection, obliterate the dead space, and cover synthetic meshes [10, 14]. The indication of rigid prosthesis or synthetic meshes for reconstruction of the chest wall is not well defined. Lardinois et al. [15] suggest the use of rigid prostheses when there are associated resections of more than 3 ribs. Bosc et al. [16] also suggest the use of flexible meshes for chest wall reconstruction when there is resection of up to 4 ribs.

Only two patients in this case series used synthetic meshes for reconstruction; none used rigid prosthesis, being extubated in the immediate postoperative period. Consequently, the use of synthetic materials for reconstruction of the chest wall was not relevant to patient outcome. More studies are needed to define the validity and indication of the use of meshes and prostheses in the reconstruction of the chest wall after sternectomy.

Besides the complexity and morbidity associated with extended sternectomy, the patient's overall survival was relevant, even for patients with advanced disease. Among patients with recurrence, therapeutic options could offer a long disease-free survival. This is a very important finding, justifying the goal of complete surgical resection of nonmelanoma skin cancer whenever possible. Certainly, this study has the bias of a retrospective case series and more studies are necessary to define the best treatment option in patients with advanced non-melanoma skin cancer invading the chest wall. But, this case series has the importance to show an option for treatment of locally advanced nonmelanoma skin cancer with good results for survival and functional preservation in a scenario where the systemic treatment for nonmelanoma skin cancer lacks further evidence of therapeutic efficiency.

## 4. Conclusions

Sternectomy as a treatment of skin tumors is a dramatic event in the evolution of this disease in its later stage, implying high morbidity for their treatment. Chronic exposure to solar radiation in our environment, associated with the cultural habit of unbuttoning shirts to the abdominal region, was an essential risk factor to the development of these tumors. However, as described, the long evolution of the disease is a determining factor in the prognosis, allowing its extension to other nearby organs, such as the sternum and lymph node metastases. Therefore, we can ascribe to difficulty of access to health services as an additional risk factor for the high prevalence of sternectomies for skin tumors in our state. However, despite the complexity and morbidity associated with sternectomy, it remains a treatment option with curative purposes for locally advanced skin cancer, improving the prognosis and overall survival of these patients.

## Figures and Tables

**Table 1:** Epidemiological profile of patients.

Patient	Age [years]	Gender	Time of disease evolution until treatment [months]	Histological diagnosis
1	66	Masculine	12-24	SSCC
2	57	Masculine	>24	SSCC
3	59	Masculine	6	SSCC
4	60	Masculine	6	SSCC
5	77	Masculine	6-12	SSCC
6	69	Masculine	>24	BCC
7	49	Masculine	>24	BCC

**Table 2:** Surgical profile.

Patient	Surgery	Type of resection	Reconstruction	Adjuvant treatment (Radiotherapy)
1	Partial sternectomy, total thyroidectomy and cervical lymphadenectomy	R0	Bilateral myocutaneous flap	Yes
2	Partial sternectomy, rib and clavicle resection and mediastinal lymphadenectomy	R0	Unilateral myocutaneous flap	Yes
3	Partial sternectomy and mediastinal lymphadenectomy	R1	Unilateral myocutaneous flap and skin graft	Yes
4	Partial sternectomy and cervical lymphadenectomy	R0	Unilateral myocutaneous flap and polypropylene mesh	No
5	Partial sternectomy, rib and clavicle resection	R0	Unilateral myocutaneous flap	Yes
6	Partial sternectomy, clavicle resection and mediastinal lymphadenectomy	R0	Unilateral myocutaneous flap	No
7	Partial sternectomy	R0	Unilateral myocutaneous flap and polypropylene mesh	No

**Table 3:** Histopathological profile of patients.

Patient	Histological type	Perineural invasion	Angiolymphatic invasion	Bone invasion	Dimension of lesion [ cm]	Staging classification
1	SSCC	Yes	Yes	Yes	7.0 x 6.5	T4aN1M0
2	SSCC	*∗*	*∗*	*∗*	*∗*	T4aN0M0
3	SSCC	Yes	Yes	Yes	7.0 x 5.8	T4aN0M0
4	SSCC	No	No	Yes	12.0 x 9.5	T4aN0M0
5	SSCC	Yes	Yes	No	4.0 x 1.0	T3N0M0
6	BCC basosquamous	No	No	Yes	8.5 x 2.2	T4aN0M0
7	BCC nodular	*∗*	*∗*	Yes	*∗*	T4aN0M0

*∗*Information not available in medical records.

**Table 4:** Overall and disease-free survival.

Patient	Overall survival from the surgery to last medical evaluation [months]	Disease free survival [months]	Recurrence site	Treatment of recurrence	State of health on the last medical evaluation
1	84	84	-	-	Cancer free
2	96	19	Unilateral axillary lymph node	Surgery (axillar lymphadenectomy)	Cancer free
3	12	12	-	-	Cancer free
4	4	4	-	-	Cancer free
5	72	27	Chest wall	Radiotherapy	Treating recurrence with radiotherapy
6	72	50	Body of sternum	Surgery (total sternectomy)	Cancer free
7	12	12	-	-	Cancer free

## Data Availability

The data used to support the findings of this study are available from the corresponding author upon request.
